# Opposing roles of microRNA Argonautes during *Caenorhabditis elegans* aging

**DOI:** 10.1371/journal.pgen.1007379

**Published:** 2018-06-21

**Authors:** Antti P. Aalto, Ian A. Nicastro, James P. Broughton, Laura B. Chipman, William P. Schreiner, Jerry S. Chen, Amy E. Pasquinelli

**Affiliations:** Division of Biology, University of California, San Diego, La Jolla, CA, United States of America; UNITED STATES

## Abstract

Argonaute (AGO) proteins partner with microRNAs (miRNAs) to target specific genes for post-transcriptional regulation. During larval development in *Caenorhabditis elegans*, Argonaute-Like Gene 1 (ALG-1) is the primary mediator of the miRNA pathway, while the related ALG-2 protein is largely dispensable. Here we show that in adult *C*. *elegans* these AGOs are differentially expressed and, surprisingly, work in opposition to each other; *alg-1* promotes longevity, whereas *alg-2* restricts lifespan. Transcriptional profiling of adult animals revealed that distinct miRNAs and largely non-overlapping sets of protein-coding genes are misregulated in *alg-1* and *alg-2* mutants. Interestingly, many of the differentially expressed genes are downstream targets of the Insulin/ IGF-1 Signaling (IIS) pathway, which controls lifespan by regulating the activity of the DAF-16/ FOXO transcription factor. Consistent with this observation, we show that *daf-16* is required for the extended lifespan of *alg-2* mutants. Furthermore, the long lifespan of *daf-2* insulin receptor mutants, which depends on *daf-16*, is strongly reduced in animals lacking *alg-1* activity. This work establishes an important role for AGO-mediated gene regulation in aging *C*. *elegans* and illustrates that the activity of homologous genes can switch from complementary to antagonistic, depending on the life stage.

## Author summary

Tiny non-coding RNAs called microRNAs (miRNAs) are broadly conserved across animal species and have established roles in regulating development, metabolism and behavior. In humans, aberrant expression or function of specific miRNAs has been associated with a wide variety of diseases, underscoring the critical role of these molecules in organismal viability. Argonaute (AGO) proteins are essential co-factors for miRNAs to regulate the expression of target genes. In *C*. *elegans* nematodes, two highly related AGOs (ALG-1 and ALG-2; Argonaute-Like Genes) play largely overlapping roles in the miRNA pathway during development. Here we report that the activities of these two AGOs diverge in aging animals, as loss of ALG-1 shortens lifespan, while loss of ALG-2 extends it. These opposite longevity phenotypes are associated with differential regulation of specific miRNAs and protein-coding genes that act in the Insulin/ IGF-1 Signaling (IIS) pathway. Furthermore, we present genetic evidence that *alg-1* and *alg-2* operate within this pathway to impact aging. In sum, our findings reveal that two related AGOs function antagonistically within the conserved insulin signaling pathway that regulates longevity.

## Introduction

As components of the microRNA (miRNA) induced silencing complex (miRISC), miRNAs use partial base pairing to tether Argonaute (AGO) and associated proteins to specific target RNAs, typically resulting in RNA destabilization [1]. Each miRNA has multiple targets and regulation of individual targets ranges from fine-tuning to robust silencing [2]. Across multicellular organisms, miRNAs play integral roles in many different pathways, and changes in miRNA expression or function have been linked to numerous human diseases, including cancer, heart ailments and neuronal pathologies [3, 4].

Target regulation by miRNAs is dependent on the availability and function of AGO proteins. Of the 25 different AGOs in *C*. *elegans*, only three appear to be dedicated to the miRNA pathway [5–7]. ALG-1 (AGO-Like Gene 1) and ALG-2 are broadly expressed and bind most miRNAs, whereas ALG-5 is restricted to the germline and associates with a small subset of miRNAs [5, 8]. The *alg-1* and *alg-2* genes encode proteins that are over 75% identical at the amino acid level and appear to share similar spatiotemporal expression patterns during embryogenesis and larval development [8, 9]. Although *alg-1* loss-of-function mutants exhibit mild to severe developmental defects, *alg-2* null mutants appear to develop normally [6, 8–10]. Furthermore, global misregulation of miRNA biogenesis and target regulation is observed in animals deficient in *alg-1* alone [10–12]. Although *alg-2* cannot fully compensate for the absence of *alg-1* during larval development, embryonic lethality is only observed when both of these AGOs are depleted [6, 8]. Additionally, loss of either *alg-1* or *alg-2* results in a reduced brood size, decreased numbers of oocytes and defects in ovulation [5, 13, 14]. Overall, ALG-1 appears to serve as the primary AGO for the miRNA pathway during development with ALG-2 contributing mostly redundant functions.

MiRNA activity is also important for the viability of adult *C*. *elegans*. Depletion of *alg-1* or *alg-2* by RNAi has been shown to reduce the lifespan of adult animals [15, 16]. Likewise, animals deficient in Pasha/ DGCR8, an RNA binding protein required for the processing of most miRNAs, are short lived [17]. Thus, the general loss of mature miRNAs or the AGOs needed for their function reduces lifespan. However, individual miRNAs have also been found to regulate nematode longevity. In some instances loss of specific miRNAs (lin-4, miR-71, miR-238, miR-246 or miR-228) has resulted in shortened lifespan, whereas in others (miR-80 or miR-239a/b) lifespan extension has been observed [18–23]. Presumably, misregulation of specific targets in the miRNA mutant backgrounds is responsible for the effects on lifespan. In the case of *lin-4* mutants, up-regulation of the *lin-14* target seems to underlie the reduced lifespan of this strain [18]. In general though, it is largely unknown how the changes in gene expression caused by loss of individual miRNAs or their AGO cofactors affect aging.

Studies in *C*. *elegans* and other short-lived model animals have revealed that organismal lifespan is shaped by several, partially distinct, genetic pathways. Reduced insulin signaling, dietary restriction, diminished mitochondrial respiration, and germline removal are all examples of conditions that increase longevity in a conserved fashion [24]. In *C*. *elegans*, the lin-4 miRNA functions within the Insulin/ IGF-1 signaling (IIS) pathway [18], miR-80 responds to dietary restriction [23], and let-7 family miRNAs promote the longevity of animals lacking germ cells [25].

In the canonical *C*. *elegans* IIS pathway, insulin-like peptides bind and activate the Insulin/ IGF-1 receptor DAF-2, which leads to a signaling cascade that ultimately phosphorylates the FOXO transcription factor DAF-16 and sequesters it from the nucleus [26]. In long-lived *daf-2* mutants, phosphorylation-mediated inhibition of DAF-16 is relieved, allowing it to enter the nucleus and induce the expression of downstream targets that promote longevity. These up-regulated genes (Class I) are considered to be under the direct regulation of DAF-16 [27, 28], whereas another set of genes (Class II), involved in growth and development, undergo down-regulation when DAF-16 is active [27, 28]. Upon increased insulin signaling, the transcription factor PQM-1 localizes to the nucleus and induces the expression of Class II genes, while DAF-16 is restricted to the cytoplasm [28]. When insulin signaling is reduced, DAF-16 enters the nucleus and promotes the expression of Class I genes; at the same time, PQM-1 exits the nucleus, effectively preventing Class II gene transcription. Overall, shifts in the balance between Class I and Class II gene expression contribute to the lifespan phenotypes of mutants in the IIS pathway [28].

In this study, we discovered that the miRNA AGOs, ALG-1 and ALG-2, have distinct expression patterns and activities in aging *C*. *elegans*. We show that during adulthood the expression of *alg-1* is rapidly down-regulated, whereas that of *alg-2* is sustained. Surprisingly, the maintenance of *alg-2* does not simply provide a replacement for *alg-1* activity. Instead, we found that the two AGOs play opposing roles during adulthood, with *alg-1* promoting longevity and *alg-2* suppressing it. We detected differential expression patterns for specific miRNAs in *alg-1* and *alg-2* mutants that were consistent with their opposite lifespan phenotypes. Although largely distinct sets of protein-coding genes were misregulated in each of the AGO mutants, they converged on the IIS longevity pathway. We observed that Class I *daf-16* targets were enriched in the genes down-regulated in *alg-1* or up-regulated in *alg-2* mutants. Consistent with these expression patterns, the extended lifespan of *daf-2*, which requires active *daf-16*, was significantly reduced in *alg-1* mutants. Moreover, we found that *daf-16* and two Class I genes, *cest-1* and *asah-1*, were required for the enhanced longevity of *alg-2* mutants. Altogether, our studies reveal opposing roles for two miRNA AGOs in the conserved IIS longevity pathway.

## Results

### Differential expression of ALG-1 and ALG-2 during adulthood in *C*. *elegans*

**Fig 1:**
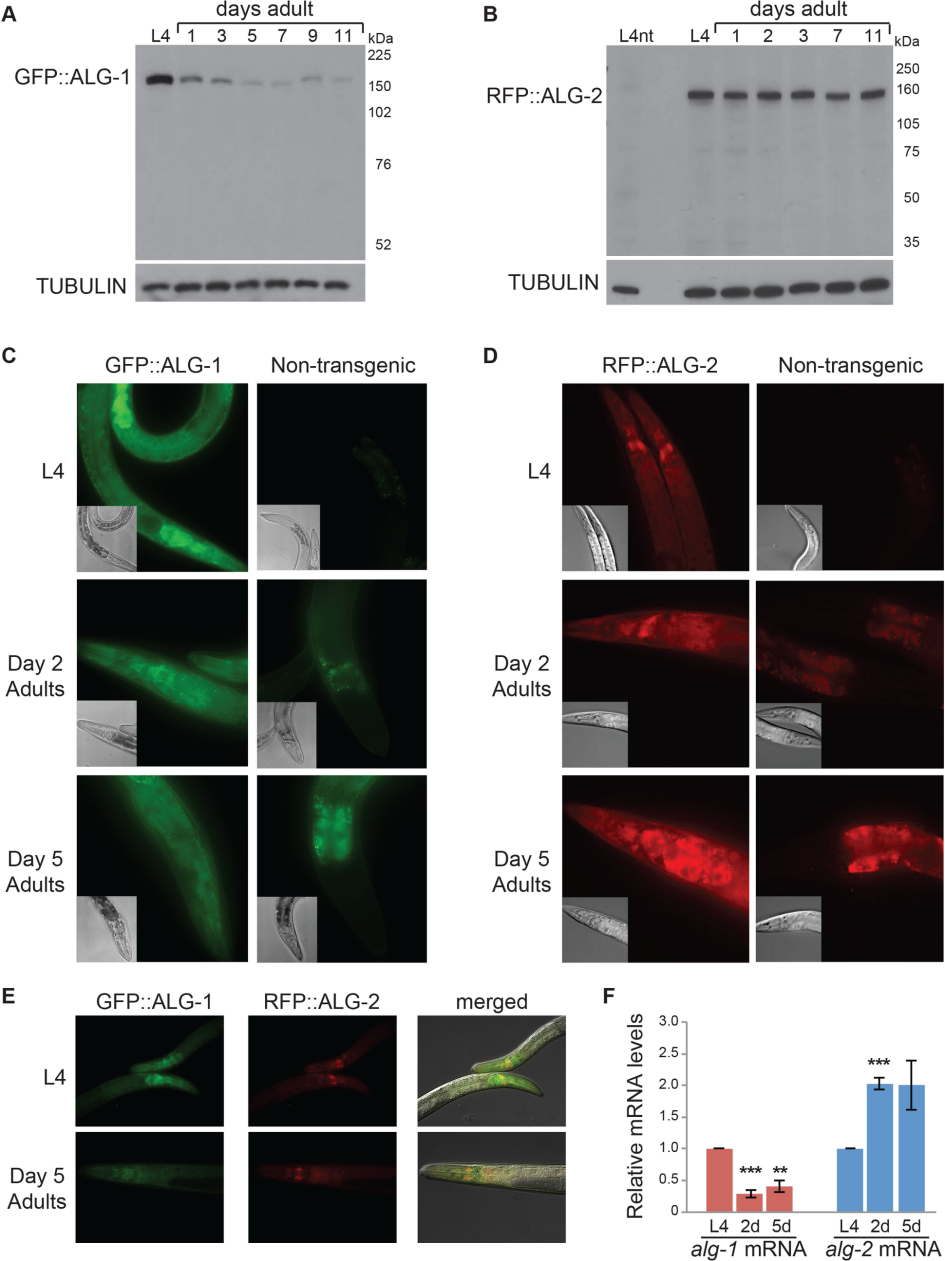
Expression of ALG-1 and ALG-2 during adulthood. **(A)** Western blot of FLAG::GFP-tagged ALG-1 in protein samples from L4 and adult stage transgenic animals. Tubulin levels serve as a loading control. **(B)** Western blot of FLAG::RFP-tagged ALG-2 in protein samples from L4 and adult stage transgenic animals. A sample from L4 stage non-transgenic (nt) animals demonstrates the specificity of the anti-FLAG antibody. Tubulin levels serve as a loading control. **(C)** Expression of endogenous ALG-1 tagged with GFP in L4, 2d and 5d adult animals visualized by fluorescence microscopy. The panels of non-transgenic animals show that auto-fluorescence contributes to the signal detected in the intestine of adults. Micrographs were captured at 40x magnification with 26 ms exposure. **(D)** Expression of endogenous ALG-2 tagged with RFP in L4, 2d and 5d adult animals visualized by fluorescence microscopy. The panels of non-transgenic animals show that auto-fluorescence contributes to the signal detected in the intestine of adults. Micrographs were captured at 40x magnification with 450 ms exposure. **(E)** Simultaneous expression of GFP::ALG-1 and RFP::ALG-2 in L4 and 5d adult animals visualized by fluorescence microscopy. Micrographs were captured at 40x magnification with equivalent exposures at L4 and 5d adult stages. **(F)** qRT-PCR analyses of *alg-1* and *alg-2* mRNA levels in adults relative to L4 stage in the *spe-9(hc88)* strain, averaged from three independent experiments. The sterile *spe-9* background was used to avoid potential signal from progeny animals. The error bars represent SEMs. **P<0.01, ***P<0.001 (t-test).

Previous studies have shown that the predominant miRNA Argonautes, ALG-1 and ALG-2, are expressed constitutively in developing *C*. *elegans*, with the highest levels detected in embryos [5, 8]. To analyze the expression of these AGOs in adult animals, we used genome editing methods [29] to tag the N-termini of the protein-coding sequences of the endogenous *alg-1* and *alg-2* genes with FLAG::GFP (ALG-1) or FLAG::RFP (ALG-2) moieties. To confirm that the tagged AGO proteins retained function, we subjected the strains to a sensitive phenotypic assay. The individual loss of *alg-1* or *alg-2* has no effect on embryonic viability. However, disruption of both genes results in nearly complete embryonic lethality [6, 8, 30]. While depletion of *alg-2* by RNAi caused highly penetrant embryonic lethality in *alg-1(gk214)* loss-of-function mutants, the same RNAi treatment had no effect on embryo viability in the GFP::ALG-1 strain, demonstrating functionality of the edited gene (S1 Fig). Likewise, *alg-1 (RNAi)* resulted in embryonic lethality in *alg-2(ok304)* mutants but not in the RFP::ALG-2 strain, confirming that the edited *alg-2* gene retained function (S1 Fig). The tagged genes produced the expected size AGO proteins, as detected with an anti-FLAG antibody (Fig 1A and 1B). While ALG-2 levels remained relatively constant from the fourth larval stage (L4) through day 11 of adulthood, ALG-1 levels decreased precipitously during adulthood (Fig 1A and 1B). The rapid decline in ALG-1 expression as animals entered adulthood was also observed using antibodies against the untagged ALG-1 protein in wildtype, as well as in the sterile *spe-9(hc88)* strain (see later for examples).

Analysis of GFP::ALG-1 and RFP::ALG-2 in live animals revealed broad spatial expression patterns for these AGOs (Fig 1C and 1D). In agreement with a previous study [8], we observed that both proteins were expressed in most somatic cells but exhibited some tissue specificity in the head region. Pharyngeal cells predominantly expressed GFP::ALG-1, while certain head neurons adjacent to the pharynx were enriched for RFP::ALG-2 (Fig 1C–1E). Consistent with the Western blot results, there was a global decline in expression of GFP::ALG-1 but not RFP::ALG-2 as the animals aged (Fig 1C–1E). We also analyzed the mRNA levels of *alg-1* and *alg-2*, using the sterile *spe-9(hc88)* background to avoid signal from progeny developing inside of adult animals. Similar to the pattern of ALG-1 protein expression, we observed strong down-regulation of *alg-1* mRNA levels in adult compared to L4 stage animals (Fig 1F), as previously reported [31]. Likewise, levels of *alg-2* mRNA were unchanged or slightly elevated in adult versus L4 animals (Fig 1F), mirroring the expression of ALG-2 protein. Altogether, these results indicate that *alg-1* and *alg-2* have distinct spatial and temporal expression patterns.

### Opposite effects of *alg-1* and *alg-2* on lifespan

**Fig 2:**
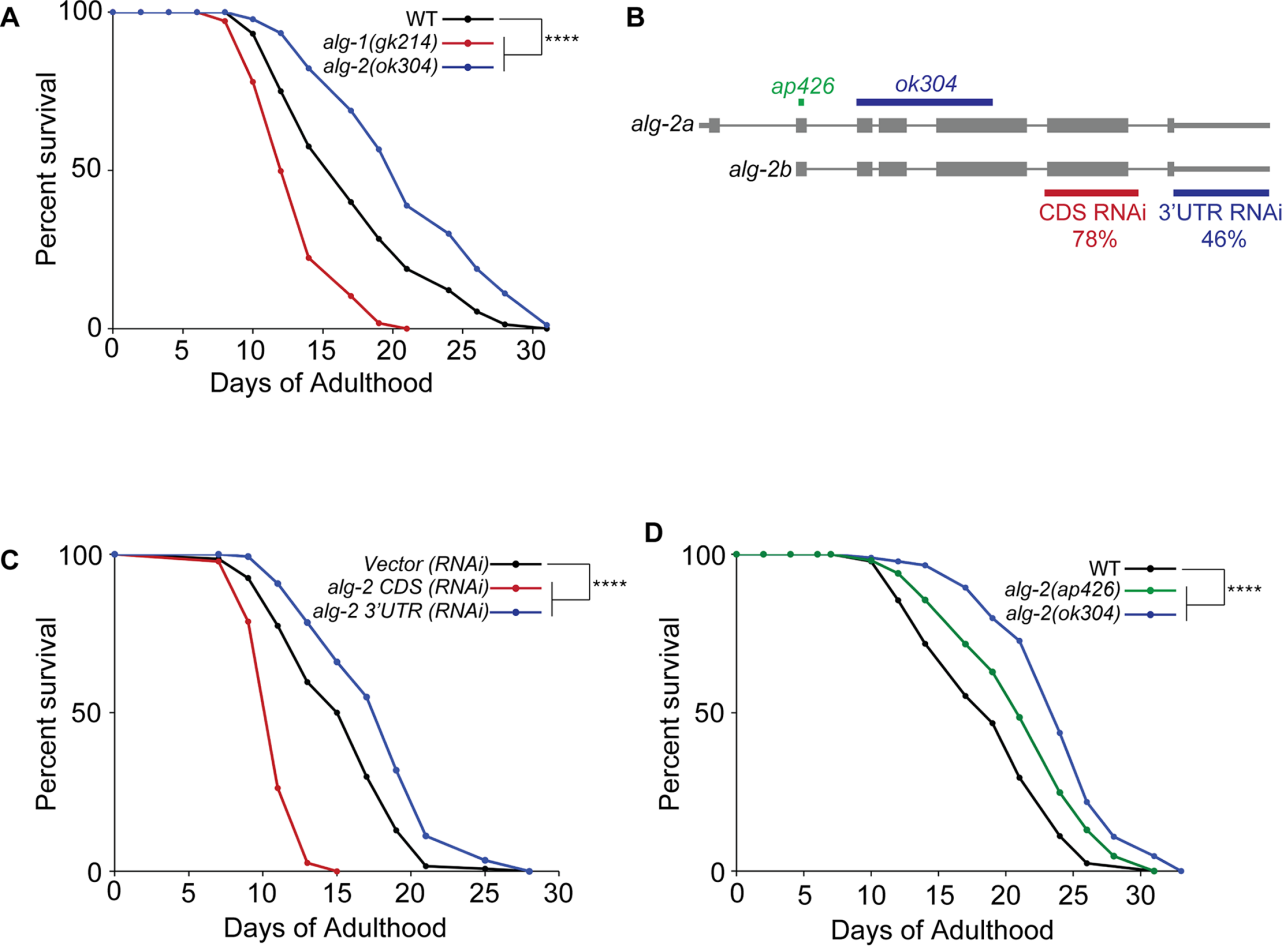
Opposite effects of *alg-1* and *alg-2* on lifespan. **(A)** Survival curves showing reduced lifespan in *alg-1(gk214)* (red) and increased lifespan in *alg-2(ok304)* (blue) compared to WT (black). ****P<0.0001 (log-rank). Statistical analyses of each replicate for all lifespan assays are shown in S1 Table. **(B)** Gene structure of *alg-2* (a and b isoforms) depicting regions targeted by RNAi and locations of genetic mutations. Sequence identity with *alg-1* in regions covered by the *alg-2* RNAi constructs is indicated by percentages. Locations of *ap426* and *ok304* deletions are depicted in green and blue, respectively (see text for details). **(C)** Survival curves showing opposite effects of RNAi targeting the coding sequence (CDS) (red) versus the 3’UTR (blue) of *alg-2* compared to vector control RNAi (black). ****P<0.0001 (log-rank). **(D)** Survival curves showing that, compared to WT (black), a new *alg-2* loss of function strain (*alg-2(ap426)*) (green) has an extended lifespan similar to that of *alg-2(ok304)* (blue). ****P<0.0001 (log-rank).

The differential regulation of *alg-1* and *alg-2* expression at the onset of adulthood prompted us to investigate potential roles for these AGOs in aging. It was previously shown that depletion of *alg-1* by RNAi treatment starting at the L4 stage leads to a shortened lifespan [15]. Consistent with this observation, we found that *alg-1(gk214)* loss-of-function mutants, which display mild developmental defects [10], have an average lifespan that is significantly shorter than that of WT (Fig 2A; S1 Table). Surprisingly, *alg-2(ok304)* loss-of-function mutants exhibited the opposite lifespan phenotype, living significantly longer than WT animals (Fig 2A; S1 Table). Initially, these results seemed to contradict a previous report that RNAi of *alg-2* shortens the lifespan of WT and long-lived *daf-2* mutant animals [16]. In that study, RNAi targeted a conserved domain in the *alg-2* coding sequence (CDS), which shares a high degree of similarity with *alg-1* (Fig 2B). To specifically repress *alg-2* alone, we created an RNAi construct for targeting the *alg-2* 3’UTR, which lacks extensive sequence homology with *alg-1*. When WT animals were subjected to RNAi targeting either the *alg-2* CDS or the 3’UTR, we observed opposite lifespan phenotypes (Fig 2C; S1 Table). In agreement with the aforementioned study [16], the original *alg-2 CDS (RNAi)* caused a significantly shortened lifespan. However, the extended lifespan of animals treated with *alg-2 3’UTR (RNAi)* was consistent with the phenotype of *alg-2(ok304)* genetic mutants. To further establish that lifespan extension was specifically associated with loss of *alg-2* activity, we created a new *alg-2* loss-of-function allele. The new *alg-2(ap426)* allele has an 8-nt deletion in the second exon (Fig 2B), which leads to a frameshift mutation and brings a premature stop codon into frame. The inability of this strain to produce viable embryos when treated with *alg-1 (RNAi)* confirmed that *alg-2(ap426)* is a new loss-of-function allele (S1 Fig). As observed for the *alg-2(ok304)* mutant, the lifespan of the *alg-2(ap426)* strain was significantly extended in comparison to WT animals (Fig 2D; S1 Table). Although both *alg-2* genetic mutant strains exhibit increased longevity, the reason for the difference in the degree of lifespan extension is unclear. In summary, our studies show that miRNA AGOs play opposing roles during aging in *C*. *elegans* and suggest that *alg-1* positively regulates lifespan, whereas *alg-2* negatively impacts it.

### Increased expression of *alg-1* is insufficient for lifespan extension

**Fig 3:**
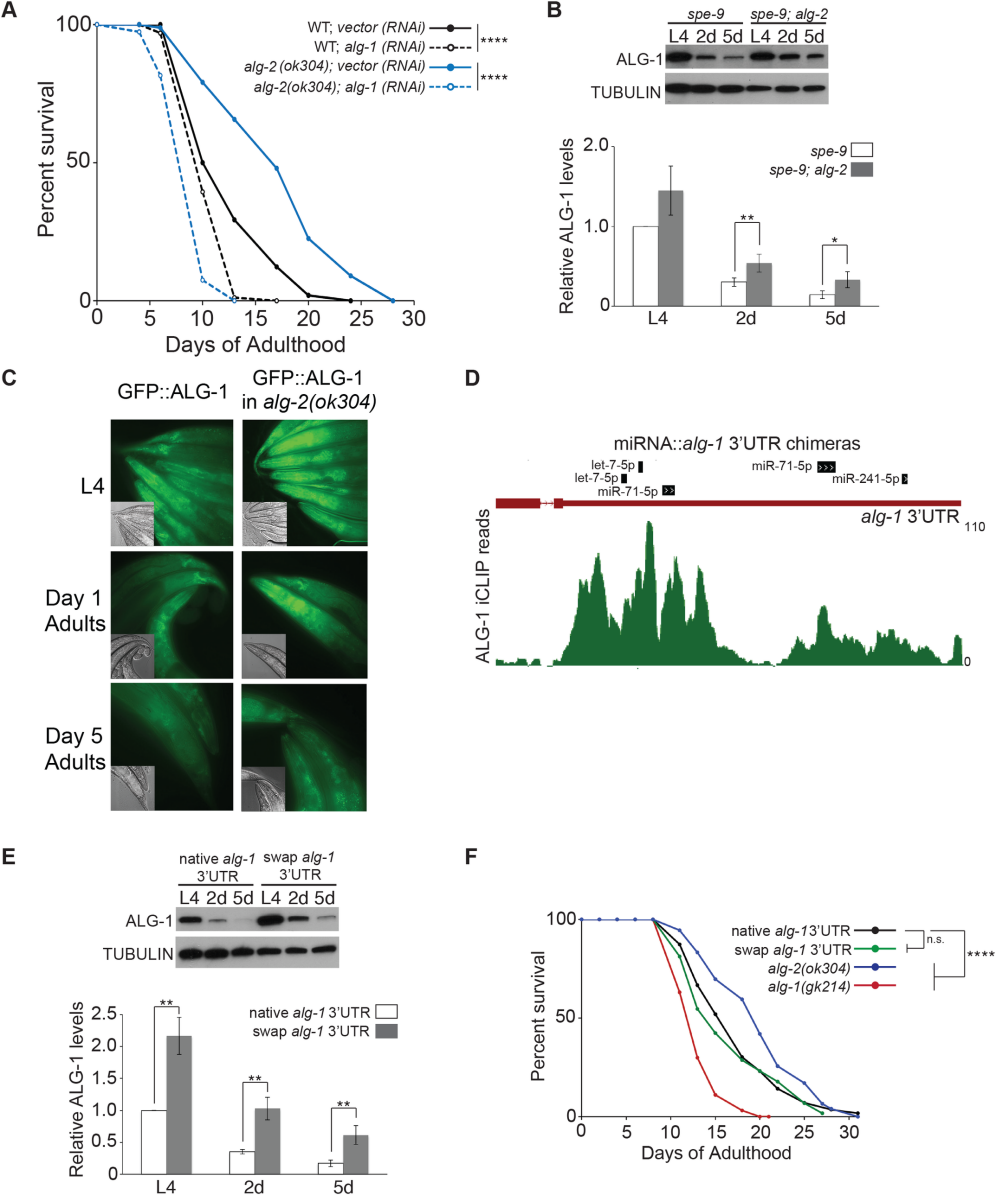
Increased *alg-1* is insufficient for lifespan extension. **(A)** Survival curves of WT (black) and *alg-2(ok304)* (blue) strains transferred to vector control (solid) or *alg-1* (dashed) RNAi at the L4 stage. ****P<0.0001 (log-rank). **(B)** Top: Western blot of ALG-1 protein levels in *spe-9(hc88)* and *spe-9(hc88); alg-2(ok304)* animals during L4 and adulthood. Bottom: Quantification of protein levels from four independent experiments. The samples were normalized to L4, and the error bars represent SEMs. **P<0.01, *P<0.05 (t-test). **(C)** Expression of endogenous ALG-1 tagged with GFP in WT versus *alg-2(ok304)* backgrounds visualized by fluorescence microscopy. Micrographs of heads & tails were captured at 40x magnification with 76 ms exposure. **(D)** Depiction of ALG-1 and miRNA binding sites in the *alg-1* 3’UTR detected by iCLIP and miRNA::target chimera analyses reported in Broughton et al., 2016 [32]. **(E)** Top: Western blot of ALG-1 protein levels expressed from the gene containing its native or a swapped 3’UTR during L4 and adulthood. Bottom: Quantification of protein levels from three independent experiments. The samples were normalized to WT L4, and the error bars represent SEMs. **P<0.01 (t-test). **(F)** Survival curves of the indicated strains, showing that replacement of the native *alg-1* 3’UTR with the Y45F10D.4 (swap) 3’UTR is not sufficient to produce a lifespan phenotype. Not significant (n.s.); ****P<0.0001 (log-rank).

Since loss of *alg-1* reduces *C*. *elegans* lifespan and expression of this AGO was observed to be down-regulated with age, we considered the possibility that *alg-2* mutants might express higher levels of ALG-1 and depend on this factor for their extended lifespan phenotype. Consistent with this idea, we found that the lifespan of *alg-2(ok304)* mutants was significantly shortened when *alg-1* was depleted by RNAi starting at the L4 stage (Fig 3A; S1 Table). The lifespan of the *alg-2(ok304)* animals subjected to *alg-1 (RNAi)* was even shorter than that of WT animals treated with *alg-1 (RNAi)*, indicating that loss of both miRNA AGOs during adulthood greatly reduces survival. Furthermore, we detected higher ALG-1 protein levels in *alg-2(ok304)* adults (Fig 3B). The sterile *spe-9(hc88)* background was used for analyses of ALG-1 protein levels to restrict detection to adult tissues. Although ALG-1 protein expression still decreased in the *alg-2* mutant background, the levels were about 2-fold higher at days 2 and 5 of adulthood compared to the ALG-1 levels in WT animals (Fig 3B). The increased expression of ALG-1 in *alg-2(ok304)* mutants was also apparent in live animals, as detected by GFP::ALG-1 fluorescence (Fig 3C).

To test if elevated ALG-1 expression would be sufficient to induce longevity phenotypes, we created a new allele of *alg-1*. Since ALG-1 targets its own 3’UTR via multiple miRNA binding sites (Fig 3D) [12, 31, 32], we reasoned that replacement of the entire endogenous 3’UTR with one considered not to be a target of the miRNA complex might result in higher expression of ALG-1. The gene *Y45F10D*.*4* was chosen because it appears to be stably expressed, is commonly used as a control gene in quantitative RT-PCR experiments, and its short 3’UTR lacks ALG-1 binding sites [12, 33, 34]. Replacement of the native *alg-1* 3’UTR with that of *Y45F10D*.*4* (swap 3’UTR) resulted in ~2-fold increase in ALG-1 protein levels (Fig 3E), which was similar to the degree of up-regulation observed at days 2 and 5 of adulthood in *alg-2(ok304)* mutants (Fig 3B). Also comparable to *alg-2* mutants, ALG-1 protein levels expressed from the edited gene still decreased as the animals transitioned from L4 to adult stages. This is likely due to transcriptional repression of *alg-1*, as a GFP-reporter driven by the *alg-1* promoter is down-regulated in adult compared to L4 animals (S2 Fig). Overall, these results indicate that 3’UTR-mediated regulation does not fully account for the decline in ALG-1 levels during adulthood.

When we performed lifespan assays, we detected no significant difference between strains with the *alg-1* gene affixed to its native 3’UTR or to the swapped 3’UTR (Fig 3F; S1 Table), which produced ALG-1 protein at levels similar to that of *alg-2(ok304)* (Fig 3B and 3E). It therefore seems unlikely that the dependence of *alg-2* on *alg-1* for increased lifespan (see Fig 3A) is simply through up-regulation of ALG-1 protein. Instead, it is possible that, while the loss of both miRNA AGOs is detrimental, ALG-1 and ALG-2 have some unique targets whose misregulation contributes to the opposite longevity phenotypes.

### Distinct miRNA and mRNA expression patterns in *alg-1* and *alg-2* mutants

**Fig 4:**
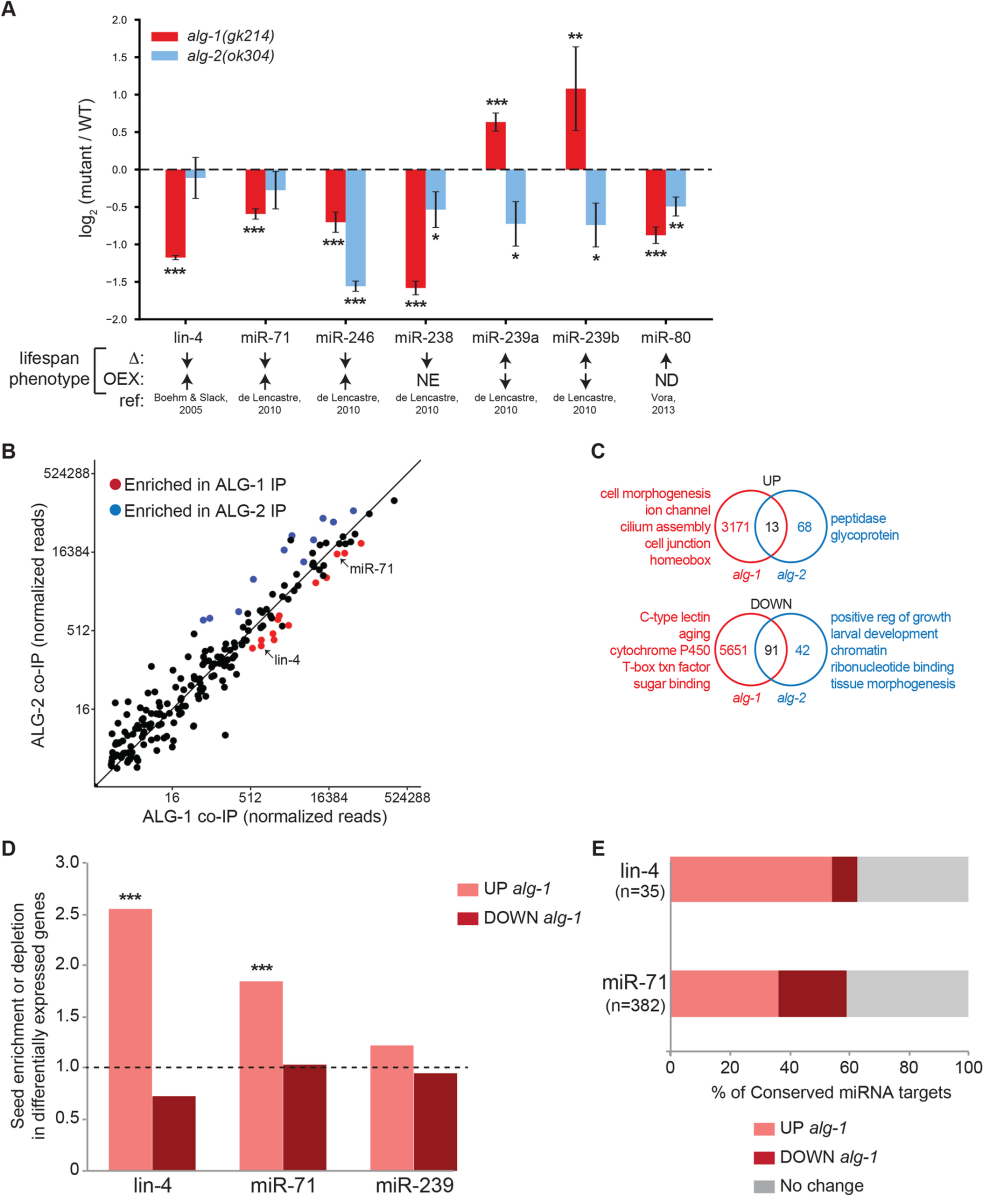
Altered miRNA and mRNA expression in *alg-1* and *alg-2* mutants. **(A)** TaqMan analyses of the indicated miRNAs in *alg-1(gk214)* (red) and *alg-2(ok304)* (blue) compared to WT animals at day 5 of adulthood, averaged from five independent experiments. The error bars represent SEMs. *P<0.05, **P<0.01, ***P<0.001 (t-test). The published lifespan phenotypes observed with reduced expression (Δ), overexpression (OEX), and corresponding references (ref) are indicated. ↑ (increased lifespan), ↓ (decreased lifespan), NE (no effect), ND (not determined). **(B)** Enrichment of specific miRNAs with ALG-1 (red) or ALG-2 (blue) detected by sequencing of small RNAs that co-immunoprecipitated (co-IP) with each AGO protein at day 5 of adulthood averaged from 2 independent experiments. See S2 Table for a complete list of miRNAs reproducibly enriched for association with ALG-1 or ALG-2. **(C)** Overlap of all genes significantly (*P*<0.05) up- or down-regulated at day 5 of adulthood in *alg-1(gk214)* (red) and *alg-2(ok304)* (blue) compared to WT animals. Overlap of down-regulated genes in *alg-1(gk214)* and *alg-2(ok304)* is more than expected by chance (*P*<0.0001, hypergeometric test). The top ranked gene ontology terms identified by DAVID analysis are listed. See S3 Table and S4 Table for complete RNA-seq and DAVID results. **(D)** Enrichment and depletion of seed pairing (nucleotides 2–7) for the indicated miRNAs with genes differentially expressed in *alg-1(gk214)* versus WT animals. The fold difference shown is in comparison to the fraction of seed-pairing sites detected in genes with unchanged expression patterns in the *alg-1* mutants. ***P<0.0001 (Chi Squared with Yates Correction). **(E)** Percent of conserved lin-4 and miR-71 targets predicted by TargetScan [36, 37] that are differentially regulated in *alg-1(gk214)*.

Previous studies have implicated several individual miRNAs as regulators of *C*. *elegans* lifespan (Fig 4A) [18, 20, 23]. To test if the expression of these aging-associated miRNAs is altered in *alg-1* and *alg-2* mutants, we analyzed their levels in RNA samples collected from WT and each of the AGO mutants at day 5 of adulthood. Day 5 was chosen because by this point the animals are mostly post-reproductive but still viable. In this panel, only two miRNAs, lin-4 and miR-71, were specifically down-regulated in *alg-1* but not *alg-2* mutants (Fig 4A). Conversely, the levels of miR-239a/b were increased in *alg-1(gk214)* and decreased in *alg-2(ok304)* (Fig 4A). These differential expression patterns in the AGO mutants are consistent with previously reported longevity phenotypes associated with these miRNAs; *lin-4* or *miR-71* mutants exhibit shortened lifespans, whereas *miR-239a/b* mutants display enhanced longevity [18, 20]. We also asked if there was preferential binding to either AGO by the aging-associated, or any other miRNAs, in day 5 adults. Through co-immunoprecipitation assays, thirteen and eleven different miRNAs were found to be predominantly associated with ALG-1 or ALG-2, respectively (Fig 4B and S2 Table). Notably, lin-4 and miR-71 were among the miRNAs enriched for binding to ALG-1 compared to ALG-2 (Fig 4B and S2 Table). This preference could underlie the reduced levels of lin-4 and miR-71 in *alg-1* mutant animals (Fig 4A), since AGO-association stabilizes mature miRNAs [35].

Based on the differences in miRNA expression and AGO-binding, we predicted that distinct sets of protein-coding genes would be misregulated in *alg-1* and *alg-2* mutants. Transcriptome profiling revealed extensive changes in gene expression in the *alg-1(gk214)* mutants compared to WT day 5 adults. We detected significant up-regulation of 3,184 and down-regulation of 5,742 genes in the *alg-1(gk214)* mutants (S3 Table). In contrast, only 81 and 133 genes were up- or down-regulated, respectively, in *alg-2(ok304)* mutants compared to WT animals (S3 Table). Notably, there was minimal overlap in genes up-regulated in both AGO mutants (Fig 4C), and each set of genes was enriched for distinct Biological Process Gene Ontology (GO) terms (Fig 4C; S4 Table). Two-thirds of the genes down-regulated in *alg-2(ok304)* mutants were also down in *alg-1(gk214)* animals. Despite this overlap, the unique down-regulated gene sets in each AGO mutant were also enriched for distinct GO terms (Fig 4C; S4 Table).

We next asked if the differential gene expression patterns might be associated with the altered miRNA levels in the *alg-1* mutants; too few genes were changed in *alg-2(ok304)* to test for enrichment or depletion of miRNA target sites. Strikingly, genes up-regulated in *alg-1(gk214)* were enriched for 3’UTR sequences that could pair to the seed region, nucleotides 2–7, of lin-4 and miR-71 (Fig 4D). While the increased levels of miR-239a/b in *alg-1* mutants were expected to result in stronger target repression, this signature was not observed in the *alg-1* down-regulated gene set (Fig 4D). When we considered only conserved miRNA targets sites predicted by the TargetScan algorithm [36, 37], over 50% of the lin-4 targets were up-regulated in *alg-1* mutants (Fig 4E). Additionally, almost twice as many conserved miR-71 targets were in the up- compared to down-regulated *alg-1(gk214)* genes (Fig 4E). There are four genes up-regulated in *alg-1* mutants that have conserved target sites for both lin-4 and miR-71 in their 3’UTRs. One of these targets, *lin-14*, has previously been shown to contribute to the shortened lifespan of *lin-4* mutant animals [18]. Taken together, the decreased expression of lin-4 and miR-71 in *alg-1* mutants likely results in the up-regulation of *lin-14* and other predicted targets of these miRNAs, which contributes to their shortened lifespan.

### Differential regulation of genes in the IIS pathway by *alg-1* and *alg-2*

**Fig 5:**
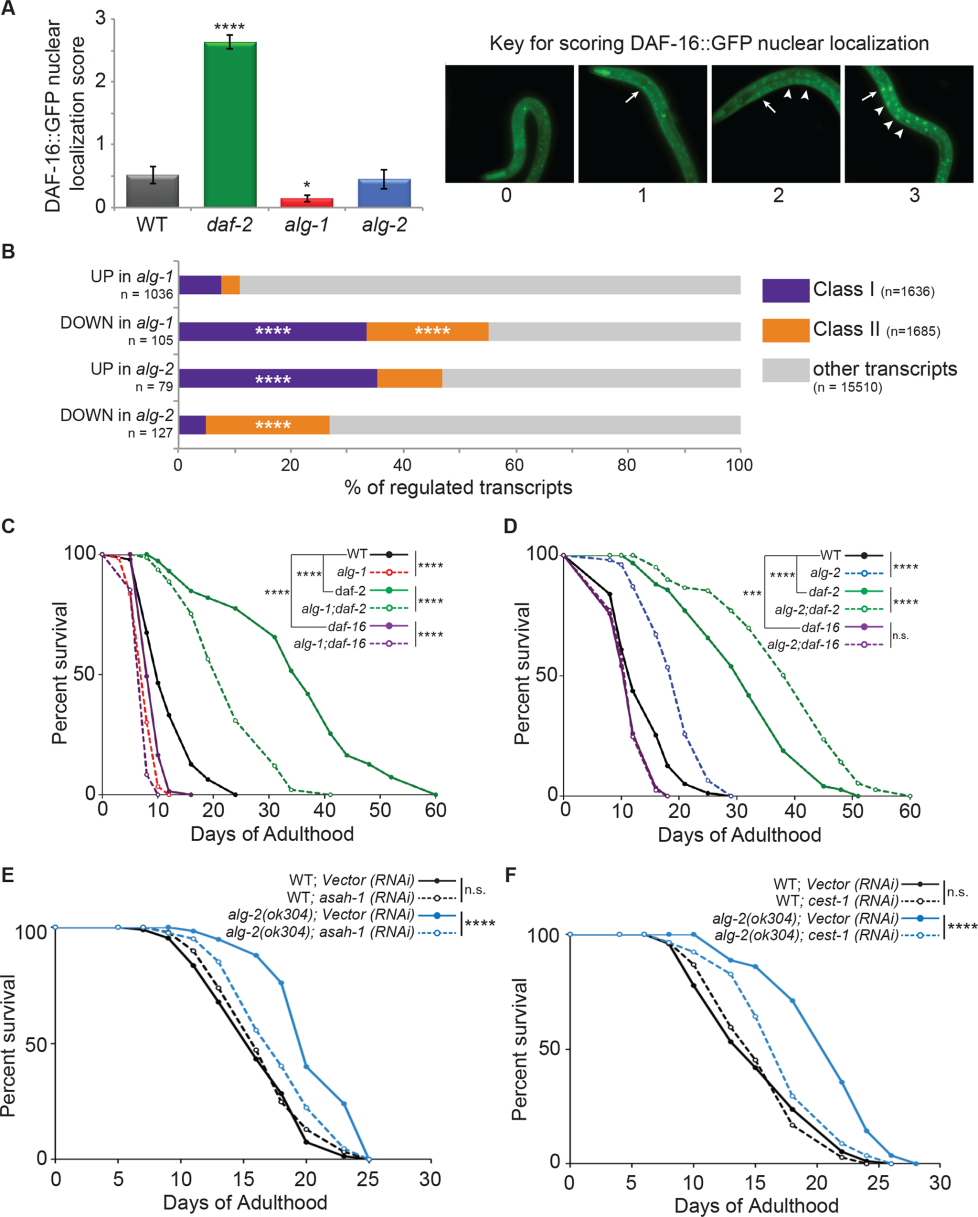
Differential regulation of the IIS pathway by *alg-1* and *alg-2*. **(A)** Left: Average DAF-16::GFP intestinal nuclear localization score for WT, *daf-2(e1370)*, *alg-1(gk214)*, *alg-2(ok304)* from four blinded scorers. The error bars represent SEMs. ****P<0.0001, *P<0.05 (t-test). Right: representative images of scoring key showing (0) no intestinal nuclear localization, (1) nuclear localization restricted to the most anterior pair of intestinal nuclei, denoted by an arrow, (2) moderate nuclear localization in the anterior pair and additional intestinal nuclei, indicated by arrowheads, and (3) strong nuclear localization in intestinal nuclei. **(B)** *The* two upper bars show the overlap of genes up- or down-regulated at least 4-fold in *alg-1(gk214)* with Class I (purple) or Class II (orange) genes [28]. The two lower bars show the overlap of all genes up- or down-regulated in *alg-2(ok304)* with Class I or II genes [28]. Enrichment: Class I genes and down in *alg-1* (****P<0.0001), Class II genes and down in *alg-1* (****P<0.0001), Class I genes and up in *alg-2* (****P<0.0001), Class II genes and down in *alg-2* (****P<0.0001) (Hypergeometric test using fractions of Class I and II genes across genome). **(C)** Survival curves of single and double mutants of the *alg-1(gk214)*, *daf-2(e1370)*, *daf-16(mu86)* strains compared to WT. ****P<0.0001 (log-rank). **(D)** Survival curves of single and double mutants of the *alg-2(ok304)*, *daf-2(e1370)*, *daf-16(mu86)* strains compared to WT. Not significant (n.s.); ****P<0.0001 (log-rank). *(E)* Survival curves of WT and *alg-2(ok304)* strains transferred to vector control or *asah-1* (*RNAi)* at the L4 stage. Not significant (n.s.); ****P<0.0001 (log-rank). *(F)* Survival curves of WT and *alg-2(ok304)* strains transferred to vector control or *cest-1* (*RNAi)* at the L4 stage. Not significant (n.s.); ****P<0.0001 (log-rank).

Loss of lin-4 or miR-71 reduces lifespan and loss of miR-239a/b extends it, at least partially through the insulin signaling pathway [18, 20]. Since these miRNAs were differentially expressed in the AGO mutants (Fig 4A), we asked if the IIS pathway would also be affected by *alg-1* or *alg-2* deficiency. The nuclear residence of the FOXO transcription factor DAF-16 is an indicator of insulin signaling levels, with reduced signaling promoting nuclear accumulation, transcription of Class I DAF-16 targets and lifespan extension [28, 38–40]. If the AGOs function upstream of DAF-16, then the prediction is that nuclear residence of DAF-16 will be reduced in *alg-1* and increased in *alg-*2 mutant backgrounds. Examination of strains expressing a DAF-16::GFP transgene that rescues the short lifespan of *daf-16(mu86)* mutants [41] revealed no obvious differences in the diffuse pattern of DAF-16 localization in the *alg-2(ok304)* background compared to WT (Fig 5A; S3 Fig). When nuclear accumulation was detected in these strains, it was restricted to the most anterior intestinal cells. In contrast, nuclear accumulation of DAF-16::GFP in multiple intestinal cells of *daf-2(e1370)* mutants was readily detected (Fig 5A; S3 Fig). Interestingly, the nuclear localization of DAF-16::GFP was significantly lower in *alg-1* mutants than in WT animals (Fig 5A; S3 Fig). These results suggest that loss of *alg-1*, but not *alg-2*, alters the nuclear residence of DAF-16.

To further explore the possibility that *alg-1* and *alg-2* differentially impact the IIS longevity pathway, we examined the expression of genes considered positive (Class I) and negative (Class II) targets of *daf-16* regulation in each of the AGO mutants at day 5 of adulthood [27, 28]. Consistent with reduced nuclear localization of DAF-16::GFP (Fig 5A), there was substantial overlap between the most strongly down-regulated genes in *alg-1(gk214)* mutants and those positively regulated by *daf-16* (Fig 5B). We observed that 33% of the genes reduced by at least 4-fold in the *alg-1(gk214)* mutants belong to Class I (Fig 5B). Some of these Class I down-regulated genes encode proteins with cytochrome P450 (*cyp-35B2*), oxidoreductase (*sodh-1*) and glutathione-S-transferase (ortholog of human *GSTP1*) activities, and RNAi depletion of these factors in WT animals shortens lifespan [27, 42]. Unexpectedly, the set of highly down-regulated genes in *alg-1(gk214)* was also enriched for Class II genes (Fig 5B). The *alg-1* up-regulated genes were not enriched for either Class, although the total number of Class I genes in this category exceeds that in the down-regulated gene set (Fig 5B). Thus, the relationship between the effect of *alg-1* on DAF-16 nuclear localization and regulation of Class I and II genes is not straightforward.

Although nuclear accumulation of DAF-16::GFP in intestinal cells was indistinguishable in *alg-2(ok304)* versus WT animals (Fig 5A), we observed a striking signature of altered *daf-16* output in the *alg-2* mutants. We found that 35% of all the genes significantly up-regulated in *alg-2(ok304)* mutants compared to WT animals were Class I DAF-16 targets (Fig 5B), including three genes (*lea-1*, *asp-3* and *cdr-6*) previously implicated in *C*. *elegans* stress tolerance or lifespan control [27, 43, 44]. Additionally, of the genes down-regulated in *alg-2* mutants, more than five times as many are considered Class II versus Class I genes (Fig 5B). Thus, *alg-2* regulates the expression, directly or indirectly, of many positive and negative targets of *daf-16* activity.

### Opposite effects of *alg-1* and *alg-2* on longevity through the IIS pathway

Since the gene expression profiles of *alg-1(gk214)* and *alg-2(ok304)* mutants indicated that the IIS pathway is perturbed in these mutants, we next asked if their altered lifespan phenotypes were dependent on key regulators of this pathway. We found that the reduced lifespan of *alg-1(gk214)* mutants was suppressed in the *daf-2(e1370)* mutant background (Fig 5C; S1 Table). However, the mean lifespan of the *alg-1(gk214); daf-2(e1370)* double mutant was about 30% shorter than that of *daf-2(e1370)* alone. Although caution must be exerted when interpreting the combined effect of incomplete loss-of-function mutations, the results suggest that *daf-2* mutants are partially dependent on *alg-1* activity for their extended lifespan phenotype. When *alg-1(gk214)* was combined with *daf-16(mu86)*, the mean lifespan of the double mutants was slightly shorter than that of either single mutant (Fig 5C; S1 Table), making it likely that the reduced lifespan of *alg-1(gk214)* mutants is not entirely through down-regulation of *daf-16* activity. This is not surprising given the large fraction of genes misregulated in *alg-1(gk214)* mutants at day 5 of adulthood, and the previous reports that loss of lin-4 or miR-71 can impact multiple longevity pathways [18–20, 22, 31].

Interestingly, the long lifespans of *alg-2(ok304)* and *daf-2(e1370)* single mutants were additive in strains harboring both mutations (Fig 5D; S1 Table). One interpretation of this result is that independent pathways contribute to the long lifespan of each mutant strain. However, the data are also consistent with the possibility that the loss of *alg-2* further weakens *daf-2* signaling, since the *daf-2(e1370)* allele is non-null. For example, it has been shown that the long lifespan of a hypomorphic *daf-2* mutant strain can be further extended by *daf-2 (RNAi)* treatment [45]. Regardless of mechanism, the long lifespan of *alg-2(ok304)* mutants was completely suppressed by the *daf-16(mu86)* mutation (Fig 5D; S1 Table). The nearly identical short lifespan curves of *daf-16* and *alg-2; daf-16* mutants indicate that the long lifespan of *alg-2(ok304)* animals is dependent on *daf-16*. Overall, these results illustrate the disparate effects of two miRNA AGOs on the regulation of gene expression and lifespan through the IIS pathway during *C*. *elegans* aging.

Since the genetic evidence indicates that *daf-16* is required for the extended lifespan of *alg-2* mutants, we asked if up-regulated Class I genes in *alg-2(ok304)* contribute to their longevity phenotype. We focused on *asah-1* (N-Acylsphingosine amidohydrolase 1; K11D2.2) because it was previously reported that RNAi of this gene partially reduced the extended lifespan of *daf-2* mutants [27] and *cest-1* (carboxyl esterase domain containing 1; T02B5.1*)* because it is the highest ranking Class I gene (13 out of 1663) up-regulated in the *alg-2* mutants [28]. We found that the extended lifespan of *alg-2(ok304)* was almost completely suppressed in animals subjected to RNAi of *asah-1* or *cest-1* starting at the L4 stage of development (Fig 5E and 5F). Notably, RNAi of *asah-1* or *cest-1*, compared to vector control RNAi, had no significant effect on the lifespan of WT animals (Fig 5E and 5F), indicating that this treatment did not generally reduce viability. These results suggest that increased expression of *asah-1*, *cest-1* and possibly other Class I targets in *alg-2* mutants contributes to the extended lifespan of these animals.

The IIS pathway also controls the ability of larval *C*. *elegans* to arrest development and enter a specialized stage called dauer [46]. Normally, entry into the dauer stage occurs in response to harsh environmental conditions. However, animals with mutations in certain *daf* (dauer formation) genes inappropriately become dauers under favorable conditions. For example, *daf-28(sa191)* mutants produce an aberrant version of the DAF-28 insulin-like peptide that reduces DAF-2 signaling and causes dauer formation during optimal culture conditions at 20°C [47, 48]. Although *alg-1(gk214)* and *alg-2(ok304)* mutants did not form dauers at 20°C (S4 Fig), we asked if loss of either of these AGOs would modify the dauer phenotype of *daf-28(sa191)* animals. In this sensitized background, dauer formation was significantly suppressed by *alg-1(gk214)* and enhanced by *alg-2(ok304)* mutations (S4 Fig). Importantly, dauer formation in the *alg-1;daf-28* and *alg-2;daf-28* mutants was entirely dependent on *daf-16* (S4 Fig). These results further demonstrate a role for the miRNA AGOs in IIS and exemplify their contrasting activities during another life stage.

## Discussion

Here we show that the miRNA AGOs, ALG-1 and ALG-2, promote opposing fates during aging in *C*. *elegans*. Given their very high degree of homology and redundant roles in embryogenesis and larval development, dichotomous activities for these proteins were unexpected. We found that loss of *alg-1* shortens and loss of *alg-2* extends lifespan. Instead of compensating for the normally diminished levels of *alg-1* in adult animals, the sustained expression of *alg-2* during adulthood seems to limit lifespan. Consistent with the contrasting phenotypic effects due to loss of *alg-1* or *alg-2*, distinct miRNAs and largely non-overlapping sets of protein-coding genes were found to be misregulated at day 5 of adulthood in these mutants. Nonetheless, the classes of differentially expressed genes in each mutant pointed to altered regulation of a common pathway–the Insulin/IGF-1 signaling (IIS) pathway. Our results indicate that *alg-1* activity positively regulates the expression of many *daf-16* target genes and contributes to the extended lifespan of *daf-2* mutants. Conversely, *alg-2* represses the expression of many *daf-16* targets to limit lifespan. Overall, this work reveals that two seemingly homologous proteins can promote opposite fates in specific biological pathways and life stages.

### Dynamic Argonaute expression patterns

As the core effector protein of miRISC, Argonaute is expected to display a broad spatiotemporal expression pattern. Previous work has shown that the *C*. *elegans* miRNA AGOs exhibit largely overlapping expression domains in most embryonic and larval tissues, which is consistent with their redundant functions at these stages [5, 8–10]. However, we observed a distinct pattern in adults where global ALG-1 levels plummeted as ALG-2 levels remained constant (Fig 1). Whereas the protein coding sequences of these AGO genes are ~75% identical, the regulatory sequences, including promoter and untranslated regions (UTRs), are highly divergent. Additionally, modENCODE data show very different transcription factor binding profiles for *alg-1* and *alg-2* [49], suggesting that these genes may be subject to distinct transcriptional control mechanisms.

The down-regulation of *alg-1* as animals enter adulthood appears to be mediated by both transcriptional and post-transcriptional mechanisms. The *alg-1* 3’UTR contains many predicted miRNA target sites and biochemical experiments have detected association of this 3’UTR with ALG-1 and specific miRNAs, including miR-71, at the L4 stage (Fig 3D) [32]. Recently, Slack and colleagues confirmed that *alg-1* is subject to regulation by miR-71 in adults; loss of this miRNA or its target sites in the *alg-1* 3’UTR resulted in increased ALG-1 levels [31]. Not surprisingly, then, exchange of the entire *alg-1* 3’UTR with one unlikely to be targeted by the miRNA complex resulted in higher levels of ALG-1 protein (Fig 3E). This increase resembled the elevated levels of ALG-1 observed in *alg-2(ok304)* adults (Fig 3B), raising the possibility that *alg-2* directly or indirectly regulates the expression of *alg-1* through its 3’UTR at this life stage. However, this pathway is only partially responsible for the down-regulation of ALG-1 in adult animals, since levels of ALG-1 still decreased in the absence of *alg-2* or the native *alg-1* 3’UTR (Fig 3B and 3E). A GFP reporter fused to only the promoter sequences of *alg-1* also displayed marked down-regulation at the adult compared to larval stages, pointing to a layer of transcriptional control for limiting *alg-1* expression in adults (S2 Fig). While the *alg-1* and *alg-2* genes have maintained a very high degree of protein sequence identity, they apparently have evolved distinct regulatory elements that drive divergent expression patterns in *C*. *elegans* transitioning from the larval to adult stages.

### Divergent roles for *alg-1* and *alg-2* in adult animals

The previously described complementary roles of *alg-1* and *alg-2* during embryogenesis and larval development are consistent with the similar expression patterns of these highly homologous proteins at these stages [5, 6, 8–10]. The opposing effects of *alg-1* and *alg-2* on lifespan seem to act specifically at adulthood, since depletion of either gene starting at the L4 stage was sufficient to produce longevity phenotypes (Figs 2C and 3A). Notably, RNAi targeting sequences in the *alg-2* 3’UTR that lacked homology with *alg-1* was necessary for observing a lifespan extension phenotype similar to that exhibited by *alg-2* loss-of-function genetic mutants (Fig 2). This illustrates that while RNAi can be useful for depleting the expression of related genes that might have redundant functions, it can also obscure potentially distinct phenotypes of individual homologs.

Presently, it is unclear if the contrasting roles of *alg-1* and *alg-2* during aging are due to differences in expression or protein function. We found that *alg-1* is strongly down-regulated in adult animals (Fig 1). Yet, genetic or RNAi-induced loss of *alg-1* shortened lifespan (Figs 2 and 3), suggesting that the residual expression of ALG-1 in WT animals is important for longevity. However, increasing ALG-1 protein levels by ~2-fold did not produce a lifespan phenotype (Fig 3E and 3F). Thus, normal aging depends on a minimal level of ALG-1, but doubling its expression does not have an obvious effect on lifespan.

Although the ALG-1 and ALG-2 proteins are predicted to be structurally very similar given their high degree of sequence identity [6, 8, 9], a few functional differences have been reported. Non-overlapping sets of genes have been found to have synthetic lethal interactions with *alg-1* or *alg-2* mutant animals [9]. This study also reported that ectopically expressed ALG-1 and ALG-2 fractionate into distinct complexes, yet associate with the same populations of miRNAs [9]. However, other studies found that some miRNAs were enriched for association with ALG-1 or ALG-2 in different stages of larval development [5, 8], consistent with our observations in adult animals (Fig 4B). Finally, through unique sequences in its N-terminal domain, ALG-1, but not ALG-2, binds the Receptor for Activated C-Kinase (RACK1) [50]. This interaction was shown to contribute to the repressive function of miRISC in *C*. *elegans* [50], although additional roles for RACK1 in miRNA biogenesis and stability have been proposed [51–53]. Some of these reported differences in *alg-1* and *alg-2* might be related to the contrasting roles of these genes during aging. However, since *alg-1* and *alg-2* functionally overlap during embryonic and larval development, there would need to be an adult-specific mechanism to convert these AGOs into proteins with opposing activities. For example, protein modifications or the expression of different binding partners restricted to adulthood could enable the regulation of distinct targets by ALG-1 and ALG-2. Our identification of miRNAs and protein-coding genes differentially regulated by *alg-1* and *alg-2* at day 5 of adulthood further illustrates the divergent activities of these AGOs during aging and points to a common pathway that could explain the opposite effects of these genes on lifespan.

### Differential effects on IIS by *alg-1* and *alg-2*

Regulation of lifespan through the IIS pathway is almost entirely dependent on the conserved transcription factor DAF-16. When IIS is low, activated and nuclear localized DAF-16 promotes the transcription of hundreds of genes, which ultimately results in animals with longer and healthier lifespans [24]. Although some individual DAF-16 targets have been shown to regulate longevity, in general the cumulative up- or down-regulation of many genes controlled by *daf-16* is likely responsible for the impressive lifespan phenotypes exhibited by mutants with reduced insulin signaling [54]. Considering the central role of *daf-16* in longevity control by IIS, the misregulation of many DAF-16 targets in *alg-1* and *alg-2* mutants suggests that these AGOs regulate lifespan, at least in part, via this pathway. Consistent with the reduced nuclear residence of DAF-16 detected in *alg-1* mutants (Fig 5A), about one-third of the most strongly down-regulated genes in these animals are classified as high confidence direct DAF-16 targets (Class I) (Fig 5B) [28]. Since individual depletion by RNAi of some of the genes on this list, such as *cyp-35B2* and *sodh-1*, results in reduced longevity [27], it is likely that decreased expression of these and many other DAF-16 targets in *alg-1* mutants contributes to their shortened lifespan.

Since we also found that *alg-1; daf-16* double mutants have slightly shorter lifespans than either mutant alone (Fig 5C), the loss of *alg-1* likely impacts other mediators of longevity. A good candidate is the heat-shock factor, *hsf-1*, which we found to be down-regulated in the *alg-1* mutant, and expression of its target *hsp-12*.*6* was three-fold lower than that detected in WT animals at day 5 of adulthood (S3 Table). The reduction of *hsf-1* or *hsp-12*.*6* levels by RNAi has been shown to decrease lifespan [55, 56], and the overexpression of *hsf-1* can extend lifespan [56], at least in part by supporting cytoskeletal integrity [57]. Thus, *alg-1* activity may affect the output of two central transcription factors in the *C*. *elegans* aging program. Additionally, given the examples of individual miRNAs influencing aging through mechanisms other than IIS [19, 23], it is likely that *alg-1* and *alg-2* function in multiple longevity pathways.

Consistent with an extended lifespan phenotype, 35% of the genes up-regulated in *alg-2* mutants are Class I DAF-16 targets (Fig 5B). Since RNAi depletion of two of the genes on this list, *asp-3* and *cdr-6*, was previously reported to decrease the lifespan of WT animals [27, 44], it is possible that even a modest increase in a combination of these and other *daf-16* targets could extend lifespan. We found that the Class I targets, *asah-1* and *cest-1*, are up-regulated in *alg-2(ok304)* mutants and are required for the extended lifespan of these animals (Fig 5E and 5F). While these genes are predicted to encode broadly conserved enzymatic proteins, little is yet known about their functions in *C*. *elegans*. CEST-1 belongs to a large family of carboxylesterases, which generally catalyze the hydrolysis of carboxylic ester substrates [58]. In mammals, some carboxylesterases function in detoxification pathways, which could be relevant for a role in longevity [59]. Previously, RNAi depletion of *asah-1* was shown to partially reduce the extended lifespan of *daf-2* mutants, further implicating it as an important player in lifespan determination through the IIS pathway [27]. ASAH-1 is homologous to human N-Acylsphingosine amidohydrolase 1 (ASAH, also known as acid ceramidase), a lipid hydrolase that converts ceramide into sphingosine and fatty acids in lysosomes [60]. In humans, loss-of-function mutations in ASAH lead to Farber’s disease, a rare inherited metabolic disorder [60]. Additionally, altered expression of ASAH has been observed in some cancers, Alzheimer’s disease, and type II diabetes, which are all diseases associated with aging [61]. Our finding that *cest-1* and *asah-1* contribute to the extended lifespan *of alg-2* mutants suggests that these predicted enzymes have longevity roles in adult animals.

The distinct set of miRNAs we found to be bound and regulated by ALG-1 and ALG-2 also reflects the divergent longevity roles of these AGOs in adult animals. Our data are consistent with a model where lin-4 and miR-71, in association with ALG-1, regulate IIS by repressing *lin-14* and other factors in this pathway, such as *daf-2*, which has a conserved miR-71 site directly bound by this miRNA at the L4 stage [32, 36, 37]. Although we did not detect up-regulation at the mRNA level for *daf-2* or any other core genes in the IIS pathway in day 5 *alg-1(gk214)* adults (S3 Table), the reduced nuclear localization of DAF-16, decreased expression of Class I genes and genetic interactions with *daf-2* imply a role for ALG-1 in restricting insulin signaling. The up-regulation of miR-239a/b in *alg-1(gk214)* may also contribute to the shortened lifespan of these animals, since deletion of this miRNA locus results in increased longevity [20]. Likewise, reduced levels of miR-239a/b in *alg-2(ok304)* may factor into their extended lifespan. While previous genetic studies have shown that *daf-16* is required for the extended lifespan of *miR-239a/b* mutants, direct targets of this miRNA that could be responsible for the longevity phenotype are yet to be identified [20].

In conclusion, our transcriptome profiling of day 5 adults revealed distinct gene expression patterns in *alg-1* and *alg-2* mutant animals. Although the differential expression of specific aging-associated miRNAs and *daf-16* target genes is consistent with the opposite lifespan phenotypes, there may be other relevant changes not detectable by RNA expression analyses. Differences in protein levels or modifications may also contribute to lifespan regulation by the AGOs. Although the full set of molecular changes induced by loss of *alg-1* or *alg-2* is yet to be revealed, our gene expression analyses point to changes in IIS output and our genetic experiments confirm opposing roles for these miRNA AGOs in the regulation of longevity and dauer formation through the IIS pathway. Overall, this study establishes an important role for AGO-mediated gene regulation in *C*. *elegans* aging and reveals divergent activities for highly related proteins during specific life stages.

## Materials and methods

### Nematode culture and lifespan analyses

*C*. *elegans* strains were cultured under standard conditions and synchronized by hypochlorite treatment [62]. Lifespan analyses were conducted at 20°C in the absence of FUdR, as previously described [63]. Embryos were plated on NGM plates containing OP50 and the first day after the L4 stage was regarded as adult day 0 (AD 0). Worms were picked on fresh food every other day until reproduction ceased, and scored for viability every 2 to 3 days. Animals that died by bagging, bursting, or crawling off the plates were censored. JMP IN 8.0 software was used for statistical analysis and *P*-values were calculated using the log-rank (Mantel-Cox) method. Statistics for all assays are shown in S1 Table. RNAi experiments were conducted by feeding the animals dsRNA-expressing bacteria, as previously described [64]. See Supplemental Materials for a list of strains and details on generation of new strains (S1 Text).

### Western blotting

Western blotting was performed as previously described [12, 65] using mouse monoclonal antibodies against tubulin (Sigma), FLAG (Sigma), or a custom rabbit polyclonal antibody against ALG-1 [12].

### qRT-PCR

Quantitative RT-PCR analyses of mRNA (SYBR Green) and miRNA (Taqman) levels were performed according to manufacturer’s instructions with the StepOnePlus and QuantStudio 3 Real-Time PCR Systems (Applied Biosystems). Levels were normalized to Y45F10D.4 for mRNAs and U18 snoRNA for miRNAs.

### RNA sequencing

Synchronized WT, *alg-1(gk214)* and *alg-2(ok304)* animals cultured at 20°C were collected at adult day 5 after removing eggs and progeny larvae. Adult *C*. *elegans* were separated from eggs and progeny on a daily basis by washing plates with M9 into conical tubes and allowing the adults to settle by gravity for a few minutes on a bench top. The supernatant containing larvae and eggs was then removed, and this process was repeated 3–7 times until eggs and larvae were no longer visible. Three independent RNA samples of each strain were prepared for RNA sequencing with the TruSeq Stranded Total RNA Library Prep Kit (Illumina) according to the Low Sample Protocol. 50-bp single-end indexed RNA sequencing libraries were prepared from 1 μg of RNA of each sample and used for sequencing on an Illumina HiSeq platform. Subsequent mapping of sequencing reads to the *C*. *elegans* genome (ce10) was performed using RNA-STAR [66]. Total read counts for each gene were then quantified using HTSeq [67]. These read counts were then input into DESeq [68] to determine log2-fold change and differential expression between the mutant and WT strains.

### Co-immunoprecipitation and small RNA sequencing

Synchronized FLAG::GFP::ALG-1 (PQ530) and FLAG::RFP::ALG-2 (PQ582) animals cultured at 20°C were collected at adult day 5 after removing eggs and progeny larvae. Samples were collected and sonicated in 100 mM NaCl, 25 mM HEPES, 250 μM EDTA, 2 mM DTT, 0.1% (w/v) NP-40, 0.1% SDS, 1X Complete Mini Protease Inhibitor (Sigma Aldrich), and 25 U/mL rRNasin (N251A). Cell debris was removed by centrifugation and lysates were incubated with anti-FLAG (F1804) bound to Protein G Dynabeads (10004D) for 1 hour at 4°C. Following co-immunoprecipitation, beads were washed as previously described [65]. RNA was isolated to use for small RNA library preparation. Two independent RNA samples of each strain were prepared for RNA sequencing with the TruSeq small RNA Library Prep Kit (Illumina). RNA sequencing libraries were prepared from 1 μg of RNA of each sample and sequenced on an Illumina HiSeq 4000. Adapter sequences were removed, and using miRDeep2 small RNA sequences were mapped to the *C*. *elegans* genome (WS261) and quantified based on miRNA annotations from miRBase release 21 [69, 70]. To identify miRNAs that were enriched with ALG-1 or ALG-2, we first calculated the normalized reads (reads per million in the library). MiRNAs with more than 1.5-fold the number of normalized reads in one Argonaute co-IP versus the other, from independent replicates, were considered enriched. MiRNAs with less than 1000 reads across the libraries were not included in the enrichment analyses. The results are summarized in S2 Table.

### Nuclear localization scoring

Synchronized HT1889 (*daf-16(mgDf50)*; *unc-119(ed3)*; lpIs14), HT1883 (*daf-16(mgDf50)*; *daf-2(e1370) unc-119(ed3)*; lpIs14), PQ585 (*alg-1(gk214)*; *daf-16(mgDf50)*; *unc-119(ed3)*; lpIs14) and PQ586 (*alg-2(ok304)*; *daf-16(mgDf50)*; *unc-119(ed3)*; lpIs14) animals cultured at 20°C were grown to L4 before being anesthetized with 1 mg/ml of Levamisole and imaged within a 20 min period at 40X magnification. Images of individual worms (n ≥ 11 for each strain) were presented to four blinded scorers who rated the degree of intestinal nuclear localization based on a provided key.

## Supporting information

S1 Table **Related to Figs 2, 3 and 5.** Statistics of all lifespan assays used in this study. (Spreadsheet uploaded separately).
(XLSX)

S2 Table **Related to Fig 4.** MiRNAs enriched in ALG-1 or ALG-2 co-immunoprecipitations, using extracts from day 5 adults. (Spreadsheet uploaded separately).
(XLSX)

S3 Table **Related to Fig 4.** Differentially expressed genes in *alg-1(gk214)* or *alg-2(ok304)* mutants compared to WT at day 5 of adulthood. (Spreadsheet uploaded separately).
(XLSX)

S4 Table **Related to Fig 4.** Functional annotation clustering analysis of differentially expressed genes in *alg-1(gk214)* or *alg-2(ok304)* mutants compared to WT at day 5 of adulthood. The Database for Annotation, Visualization and Integrated Discovery (DAVID) was used to identify enriched biological processes in the different gene sets [71, 72]. (Spreadsheet uploaded separately).
(XLSX)

S5 Table **Related to Figs 1, 3, 4, 5, S3 and S4.** Numerical data underlying graphs and summary statistics. (Spreadsheet uploaded separately).
(XLSX)

S1 Fig Functionality of engineered *alg-1* and *alg-2* alleles. Percent of embryos that developed into viable larvae at 20°C for the indicated strains and conditions.
(PDF)

S2 Fig Transcriptional regulation of *alg-1*. Expression of GFP fused to the *alg-1* promoter (BC-12839) visualized by fluorescence microscopy at L4 and adult day 2. Micrographs were captured at 10x magnification with equivalent exposure times.
(PDF)

S3 Fig DAF-16::GFP nuclear localization. Independent replicate showing average DAF-16::GFP intestinal nuclear localization score for WT, *daf-2(e1370)*, *alg-1(gk214)*, *alg-2(ok304)* from four blinded scorers. The error bars represent SEMs. ****P<0.0001, *P<0.05 (t-test).
(PDF)

S4 Fig Loss of *alg-1* or *alg-2* differentially affects dauer formation. Percent of animals that underwent dauer arrest at 20°C in the indicated strains. Shown are averages of three independent experiments and the error bars represent SEMs. ****P<0.0001 (Fisher’s exact test).
(PDF)

S1 Text Supplementary materials and methods. Includes strains, CRISPR genome editing details and primer sequences used in this study.
(PDF)
