# Supplementary material for: Opposing roles of microRNA Argonautes during Caenorhabditis elegans aging
Source: PLoS Genet. 2018 Jun 21;14(6):e1007379. doi: 10.1371/journal.pgen.1007379 (PMC6013023; doi:10.1371/journal.pgen.1007379)

S1 Fig. Functionality of engineered *alg-1* and *alg-2* alleles. Percent of embryos that developed into viable larvae at 20°C for the indicated strains and conditions.

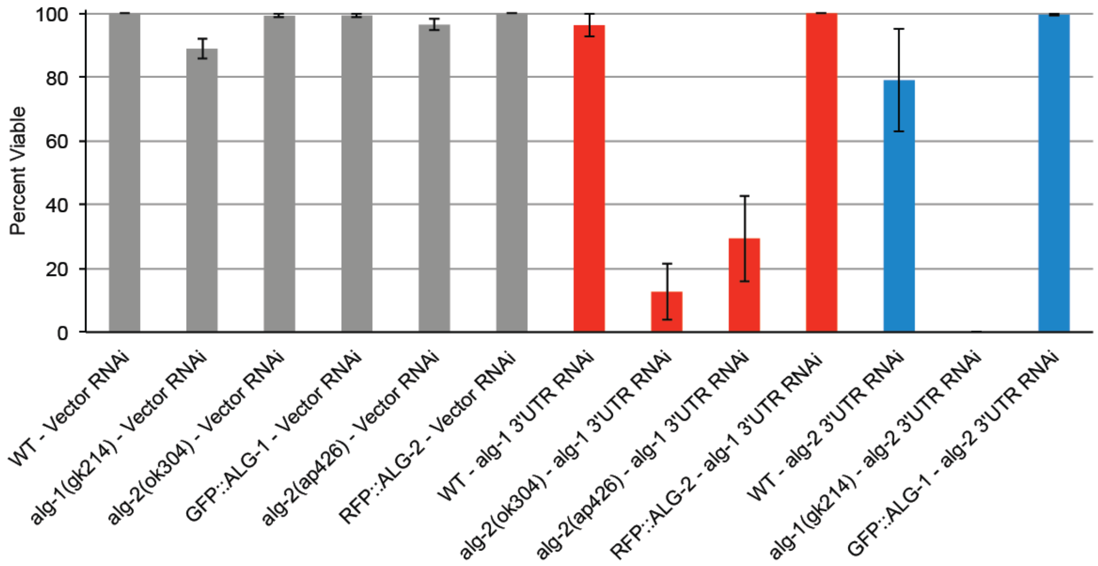

Supplement: S1 Fig — Percent of embryos that developed into viable larvae at 20°C for the indicated strains and conditions. (PDF) [file pgen.1007379.s006.pdf]
