# Supplementary material for: Opposing roles of microRNA Argonautes during Caenorhabditis elegans aging
Source: PLoS Genet. 2018 Jun 21;14(6):e1007379. doi: 10.1371/journal.pgen.1007379 (PMC6013023; doi:10.1371/journal.pgen.1007379)

S2 Fig. Transcriptional regulation of *alg-1*. Expression of GFP fused to the *alg-1* promoter (BC-12839) visualized by fluorescence microscopy at L4 and adult day 2. Micrographs were captured at 10x magnification with equivalent exposure times.

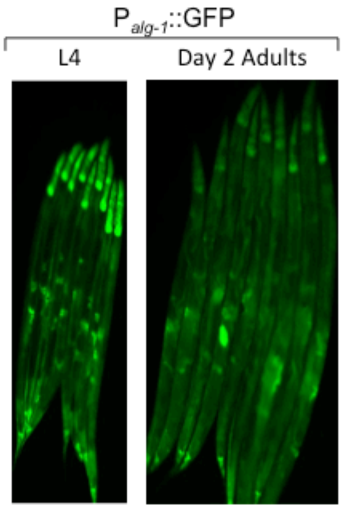

Supplement: S2 Fig — Expression of GFP fused to the alg-1 promoter (BC-12839) visualized by fluorescence microscopy at L4 and adult day 2. Micrographs were captured at 10x magnification with equivalent exposure times. (PDF) [file pgen.1007379.s007.pdf]
