# Supplementary material for: Opposing roles of microRNA Argonautes during Caenorhabditis elegans aging
Source: PLoS Genet. 2018 Jun 21;14(6):e1007379. doi: 10.1371/journal.pgen.1007379 (PMC6013023; doi:10.1371/journal.pgen.1007379)

S3 Fig. DAF-16::GFP nuclear localization. Independent replicate showing average DAF-16::GFP intestinal nuclear localization score for WT, *daf-2(e1370), alg-1(gk214)*, *alg-2(ok304)* from four blinded scorers. The error bars represent SEMs. *****P*<0.0001, **P*<0.05 (t-test).

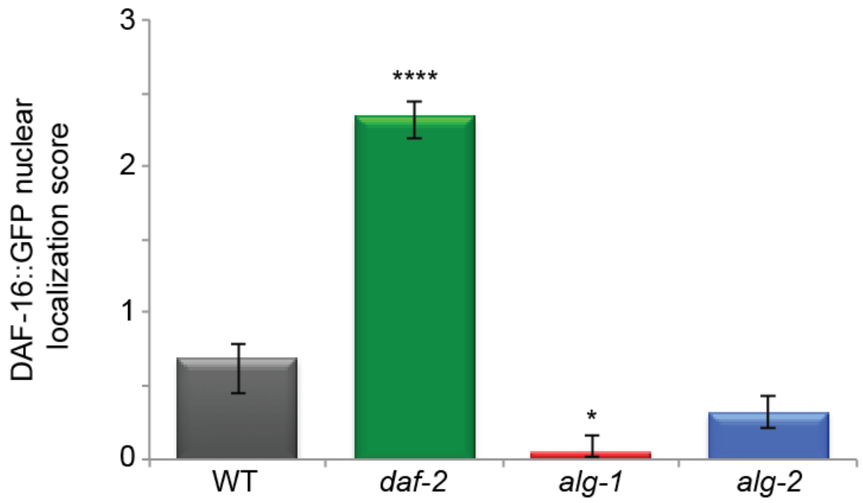

Supplement: S3 Fig — Independent replicate showing average DAF-16::GFP intestinal nuclear localization score for WT, daf-2(e1370), alg-1(gk214), alg-2(ok304) from four blinded scorers. The error bars represent SEMs. ****P<0.0001, *P<0.05 (t-test). (PDF) [file pgen.1007379.s008.pdf]
