# Supplementary material for: Opposing roles of microRNA Argonautes during Caenorhabditis elegans aging
Source: PLoS Genet. 2018 Jun 21;14(6):e1007379. doi: 10.1371/journal.pgen.1007379 (PMC6013023; doi:10.1371/journal.pgen.1007379)

S4 Fig. Loss of *alg-1* or *alg-2* differentially affects dauer formation. Percent of animals that undergo dauer arrest at 20°C in the indicated strains. Shown are averages of three independent experiments and the error bars represent SEMs. ****P<0.0001 (Fisher's exact test)

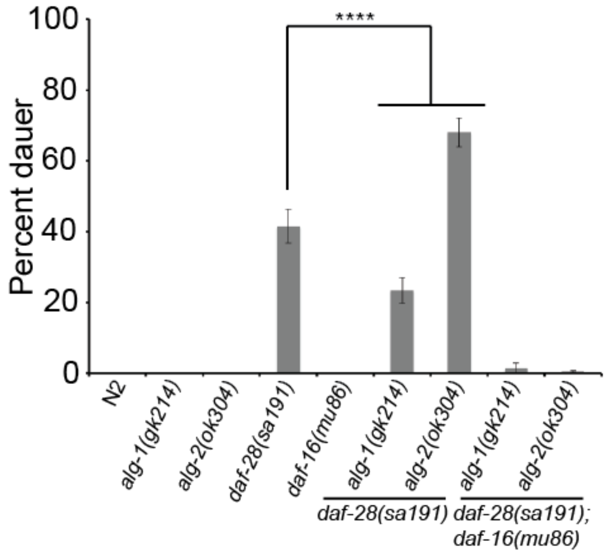

Supplement: S4 Fig — Percent of animals that underwent dauer arrest at 20°C in the indicated strains. Shown are averages of three independent experiments and the error bars represent SEMs. ****P<0.0001 (Fisher’s exact test). (PDF) [file pgen.1007379.s009.pdf]
