# Supplementary material for: Opposing roles of microRNA Argonautes during Caenorhabditis elegans aging
Source: PLoS Genet. 2018 Jun 21;14(6):e1007379. doi: 10.1371/journal.pgen.1007379 (PMC6013023; doi:10.1371/journal.pgen.1007379)
Supplement: S1 Text — Includes strains, CRISPR genome editing details and primer sequences used in this study. (PDF) [file pgen.1007379.s010.pdf]

S1 Text

SUPPLEMENTAL EXPERIMENTAL PROCEDURES

Nematode culture and strains

Most strains were maintained at 15°C or 20°C, and experiments with the temperature-sensitive sterile strains *spe-9(hc88)* (and derivatives thereof) were performed at 25°C. Worm strains: Bristol N2 (WT), VC446 (*alg-1(gk214)*), WM53 (*alg-2(ok304)*), PQ530 (*alg-1(ap423 [3xflag::gfp::alg-1])*), PQ582 (*alg-2(ap431 [3xflag::mKate2/rfp::alg-2])*), PQ583 (*alg-1(ap423 [3xflag::gfp::alg-1]); alg-2(ap431 [3xflag::mKate2/rfp::alg-2])*), PQ535 (*alg-1(ap428 [alg-1::control (Y45F10D.4) 3'UTR])),* PQ567 ((*alg-2(ap426)*), BA671 (*spe-9(hc88)*), CF1041 (*daf-2(e1370)*), CF1038 (*daf-16(mu86)*), JT191 (*daf-28(sa191)*), BC12839 (P_{alg-1}::GFP). The following *C. elegans* strains were generated through crosses: PQ504 (*daf-2(e1370); alg-1(gk214)*), PQ505 (*daf-2(e1370); alg-2(ok304)*), PQ506 (*daf-16(mu86); alg-1(gk214)*), PQ507 (*daf-16(mu86); alg-2(ok304)*), PQ524 (*spe-9(hc99); alg-1(gk214)*), PQ525 (*spe-9(hc99); alg-2(ok304)*), PQ574 (*daf-28(sa191); alg-1(gk214)*), PQ575 (*daf-28(sa191); alg-2(ok304)*), PQ579 (*daf-28(sa191); daf-16(mu86); alg-1(gk214)*), PQ580 (*daf-28(sa191); daf-16(mu86); alg-2(ok304)*), HT1889 (*daf-16(mgDf50); unc-119(ed3)*; IpIs14 [daf-16f::GFP + unc-119(+)]), HT1883 (*daf-16(mgDf50); daf-2(e1370) unc-119(ed3)*; IpIs14 [daf-16f::GFP + unc-119(+)]), PQ585 (*alg-1(gk214)*; *daf-16(mgDf50)*; *unc-119(ed3)*; IpIs14 [daf-16f::GFP + unc-119(+)]), PQ586 (*alg-2(ok304)*; *daf-16(mgDf50)*; *unc-119(ed3)*; IpIs14 [daf-16f::GFP + unc-119(+)]).

Genome editing to produce tagged and modified endogenous genes

We used CRISPR/Cas9 genome editing methods to generate novel worm strains (Tzur et al. 2013). To make the PQ530 strain (*alg-1(ap423 [3xflag::gfp::alg-1])*), young adult N2 animals were injected with the following plasmids: 33ng/µL pJB14 (PU6:sgRNA targeting start codon of ALG-1 isoform B), 18ng/µL pCFJ104 (Pmyo-3::mCherry::unc-54; a gift from Erik Jorgensen; Addgene plasmid #19328), 2ng/µL

pCFJ90 (Pmyo-2::mCherry::unc-54utr; a gift from Erik Jorgensen; Addgene plasmid #19327), 37ng/µL *Peft-3*::*cas-9*::*tbb-2*_3'UTR, 10ng/µL Palg-1::3xFLAG::GFP::alg-1 PCR product. Injected animals were allowed to reproduce at 20°C for 3-4 days and F1 progeny expressing co-injection markers were singled to new plates and allowed to grow at 15°C for one week. Populations of F2 and F3 animals were genotyped for homologous recombination of 3xFLAG::GFP at the N-terminal of ALG-1 by PCR (primers A2855 and A1001). A successful integrant was backcrossed 4x to N2 to generate PQ530.

To make the PQ582 strain (*alg-2(ap431 [3xflag::mKate2/rfp::alg-2])*), young adult N2 animals were injected with the following plasmids: 10ng/µL pJB54 (3xFLAG::mKate2::ALG-2 homologous recombination template based on pDD285 (a gift from Bob Goldstein, Addgene plasmid #66826)), 50ng/µL pJB53 (Cas9 plasmid with ALG-2 specific sgRNA, a modification of pJW1219 (a gift from Jordan Ward, Addgene #61250)), 10ng/µL pGH8 (pRAB-3::mCherry::unc-54utr, a gift from Erik Jorgensen, Addgene plasmid #19359, pCFJ104 (Pmyo-3::mCherry::unc-54utr; a gift from Erik Jorgensen; Addgene plasmid #19328), 2.5ng/µL pMyo2::tdTomato. Isolation of recombinants was performed as described in Dickinson, et al. 2015 (Dickinson et al. 2015). Briefly, injected animals were grown at 25°C for 3 days. Plates were then flooded 1mL of 3mg/mL HygromycinB. Plates were returned to 25°C for 3 days. Non-glowing roller, Hyg resistant worms were singled to new plates. L1 larvae from plates with 100% non-glowing, roller worms were moved to new plates and heat shocked at 34°C for 4hrs to remove the cassette. Wild-type worms post heat shock were selected and screened by PCR for integration of 3xFLAG::mKate2 at the ALG-2 locus. Strain was then backcrossed 2x to N2 to generate PQ582.

For generating the PQ535 strain (*alg-1(ap428 [alg-1::control (Y45F10D.4) 3'UTR]))*, young adult N2 animals were injected with the following plasmids: 80ng/µL each of pAA106 and pAA107 (PU6:sgRNA targeting ALG-1 3'UTR), 4ng/µL pIN01 (P*myo-2*-TdTomato; a gift from Dr. Andrew Dillin), 170ng/µL *Peft-3*::*cas-9*::*tbb-2*_3'UTR, 9 ng/µL IR101 (P*rps-0*_sh::*HygR*_CeOPT::*gpd-2/gpd-3*::*mCherry*::*unc-54*_3'UTR; a gift from Dr. Jason Chin), and 60ng/µL of the recombination template plasmid pAA108 (contains the entire *Y45F10D.4* 3'UTR flanked by *alg-1* left and right recombination arms). To make the PQ567 strain (*alg-2(ap426)*), young adult N2 animals were injected with the following plasmids: 25ng/µL

each of 2 sgRNAs targeting the second exon of *alg-2*, 50ng/µL *Peft-3*::*cas-9*::*tbb-2*_3'UTR, 2.5ng/µL *Pmyo-2*::*tdTomato*, and 25ng/µL IR101. The injected animals were grown at 15°C for 2-3 days, after which the plates were flooded with 3mg/mL HygromycinB to achieve a final concentration of 0.3mg/mL and returned to 15°C for 2 days. HygromycinB resistant animals were moved to single plates, allowed to lay eggs, and genotyped to test for a change in the 3'UTR using primers A3203, A3184, and A3185 (PQ535). The potential PQ567 animals were genotyped for a change in the size of the targeted exon of *alg-2* using primers flanking the region targeted by the two sgRNAs (A3534 and A3535). A successful integrant (PQ535), and a line with a frameshift mutation resulting from an 8 nt deletion in the second exon (PQ567) were each backcrossed 4x to N2.

RNAi construct targeting the *alg-2* 3'UTR

The construct to produce specific dsRNA against *alg-2* was generated by amplifying 961bp of *alg-2* 3'UTR using primers A3374 and A3375, and amplifying L4440 with A3370 and A3371. The *alg-2* PCR product was cloned into the L4440 PCR product by USER cloning (Nour-Eldin et al. 2010). The resulting plasmid was sequenced and transformed into HT115 bacteria.

Primers

Primer	Sequence	Description
A14	GAGTCGTCTAACGTCGCCTCAAACG	Genotyping primer for alg-1(gk214)
A16	CTCGCGGGTGTACATATTGCGCTTTCC	Genotyping primer for alg-1(gk214)
A110	CAAGTGGACCGATTAGTTCGACG	Genotyping primer for alg-1(gk214)
A28	ATCCGCGGAGAGCTCAGCCTTGCT	Genotyping primer for alg-2(ok304)
A2579	GGTGGAGGCCGCGAAGTATGGTTTGG	Genotyping primer for alg-2(ok304)
A2580	TAGGCAAGCCCAGAAACTTTAGCTCCAG	Genotyping primer for alg-2(ok304)
A3061	GAAGGAATTTGAAGGGTTTCGC	Genotyping primer for spe-9(hc88)
A3062	TGAAGATGTGGTAGATTGGAAGG	Genotyping primer for spe-9(hc88)
A2946	GGAGGAACGTCGGACGATCCCGAGG	Genotyping primer for daf-2(e1370)
A2966	GCGTATGATGCCTGTTCGATGGATG	Genotyping primer for daf-2(e1370)
A2948	GAAGAAGACGACGACAAGACAGG	Genotyping primer for daf-16(mu86)
A2967	GGATCGTTCACGTGTACGCCG	Genotyping primer for daf-16(mu86)
A2968	GAACGAGTGATAGAGATAAGGTG	Genotyping primer for daf-16(mu86)
A3534	CAGAAATGCCCGGCGATTCG	Genotyping primer for alg-2(ap426)
A3535	CACATCGAGCCAAACTCAGATTTAAGTC	Genotyping primer for alg-2(ap426)
A3642	GTACTCGTCCTCTCCGTCTCG	Genotyping primer for daf-28(sa191)
A3643	GTGTAGTACTTACGTGGTTCACAGG	Genotyping primer for daf-28(sa191)
A3456	AGGCAGCCGATTACCTCGCTG	Genotyping primer for mKate2::3xFLAG::Alg-2
A3633	CAAACTAACCTTGGCTCGC	Genotyping primer for mKate2::3xFLAG::Alg-2
A3169	ATCTAAAUGTTTAGCAAAGTACATGACGTTGTGTTTTAGAGCTAGAAAT	sgRNA1 for PQ535
A3170	ATTTCTAGCUCTAAAACACAACGTCATGTACTTTGCTAAACATTTAGAT	sgRNA1 for PQ535
A3171	ATCTAAAUGTTTATCAGATATTCATATACCCAGTTTTAGAGCTAGAAAT	sgRNA2 for PQ535
A3172	ATTTCTAGCUCTAAAACTGGGTATATGAATATCTGATAAACATTTAGAT	sgRNA2 for PQ535
A3173	GGCTTAAUCAGCAATGACTGAAGTCAAGG	alg-1 LRA for PQ353
A3174	ATTCCTAGTTUAAGCAAAGTACATGACGTTGTTGG	alg-1 LRA for PQ353
A3175	ACCAAAATUTGTTATATTTTTTTTGTAAAAAATTTTCAGAATGC	alg-1 RRA for PQ353
A3176	GGTTTAAUCGAAACTAATAGACAATTTTTCTAGATC	alg-1 RRA for PQ353
A3177	AAACTAGGAAUGCCTGAAGAATGTGTAATAG	Y45F10D.4 3’UTR for PQ535
A3178	AATTTTGGUAAAAAAGCTCTTTTATATATAGCG	Y45F10D.4 3’UTR for PQ535
A3185	CTCTTTTATATATAGCGATTTCTATAACG	Genotyping primer for PQ535
A3203	GACTGGTCAAACTATTGAGTGC	Genotyping primer for PQ535
A2855	CCAGGGAGAACAACCACTACAAG	Genotyping primer for PQ530
A1001	GAGAGACCACATGGTCCTTCTTG	Genotyping primer for PQ530
A3534	CAGAAATGCCCGGCGATTCG	Genotyping primer for PQ567
A3535	CACATCGAGCCAAACTCAGATTTAAGTC	Genotyping primer for PQ567
A3370	ATCAGAUCTGCCGGTCTCCCTATAG	L4440 for RNAi
A3371	ATCAAGCUTATCGATACCGTCGACC	L4440 for RNAi
A3374	ATCTGAUGTCTCCAACTACGGATTCTCTGC	alg-2 3'UTR for RNAi
A3375	AGCTTGAUCGGAATCTATTACGGAGGTGGATATG	alg-2 3'UTR for RNAi
A2906	CGAGAACCCGCGAAATGTCGGA	pPCR primer for Y45F10.4
A2907	CGGTTGCCAGGGAAGATGAGGC	pPCR primer for Y45F10.4
A2874	AACCACAGAGCGCAACATCGTC	pPCR primer for alg-1
A2875	GTGGCTCCAGATGTTGGAACAACTTG	pPCR primer for alg-1
A2920	CTACGGATTCTCTGCCATTTCACAGC	pPCR primer for alg-2
A2921	GGGGAAGGAAAAACATGAAAATCTCAGGG	pPCR primer for alg-2

SUPPLEMENTAL REFERENCES

Dickinson DJ, Pani AM, Heppert JK, Higgins CD, Goldstein B. 2015. Streamlined Genome Engineering with a Self-Excising Drug Selection Cassette. *Genetics* **200**: 1035-1049.

Nour-Eldin HH, Geu-Flores F, Halkier BA. 2010. USER cloning and USER fusion: the ideal cloning techniques for small and big laboratories. *Methods Mol Biol* **643**: 185-200.

Tzur YB, Friedland AE, Nadarajan S, Church GM, Calarco JA, Colaiacovo MP. 2013. Heritable custom genomic modifications in Caenorhabditis elegans via a CRISPR-Cas9 system. *Genetics* **195**: 1181-1185.
